# The Impact of Rearing Salinity on Flesh Texture, Taste, and Fatty Acid Composition in Largemouth Bass *Micropterus salmoides*

**DOI:** 10.3390/foods11203261

**Published:** 2022-10-19

**Authors:** Xuedi Du, Weiwei Zhang, Jie He, Mengjie Zhao, Jianqiao Wang, Xiaojing Dong, Yuanyuan Fu, Xudong Xie, Shuyan Miao

**Affiliations:** 1 Laboratory of Aquaculture Nutrition and Feed, College of Animal Science and Technology, Yangzhou University, Yangzhou 225009, China; 2 Department of Marine Medicines and Biological Products, Ningbo Institute of Oceanography, Ningbo 315832, China; 3 Zhenjiang Xinrun Agricultural Development Co., Ltd., Zhenjiang 212100, China

**Keywords:** salinity, flesh quality, texture profile, flavor compounds, taste, largemouth bass

## Abstract

It is of great significance for the aquaculture industry to determine how rearing salinity impacts fish flesh quality. In the present study, largemouth bass was cultured in different salinities (0%, 0.3%, 0.9%) for 10 weeks, and the effect on flesh texture, flavor compounds, taste, and fatty acid composition was evaluated. We show that rearing salinity not only increased flesh water-holding capacity, but also enhanced muscle hardness, chewiness, gumminess, and adhesiveness, which was consistent with the finding in the shear value test. Morphology analysis further revealed that the effect of salinity on flesh texture was probably related to changes in myofibril diameter and density. As for the taste of the flesh, water salinity improved the contents of both sweet and umami amino acids, and reduced the contents of bitter amino acid. Meanwhile, the content of IMP, the dominant flavor nucleotide in largemouth bass muscle, was significantly higher in the 0.9% group. Interestingly, electronic-tongue analysis demonstrated that the positive effect of salinity on flavor compounds enhanced the umami taste and taste richness of flesh. Moreover, rearing salinity improved the contents of C20: 5n-3 (EPA) and C22: 6n-3 (DHA) in back muscle. Therefore, rearing largemouth bass in adequate salinity may be a practical approach to improving flesh quality.

## 1. Introduction

Fish flesh quality is a complex trait for aquatic products and an important index in aquaculture and processing [1]. Generally, flesh quality depends on at least the following three aspects: the content of detectable nutrients, texture, and taste. Flesh quality can also be affected by the microbiological changes in fish during storage and processing [2]. To date, most studies focusing on the effects of dietary formulation and rearing conditions upon flesh quality have placed their emphasis on the contents of critical nutrients, for example, the content of polyunsaturated fatty acid (PUFA) [3,4,5].

As a highly important environmental factor for aquatic animals, ambient salinity profoundly influences physical and chemical properties of aquatic animal [6,7]. In rainbow trout, for example, salinity (0.17%) showed a significant effect upon the content of total monounsaturated fatty acid (MUFA) and total n-3 polyunsaturated fatty acid (n-3 PUFA) in gonad and liver tissues [8]. Moreover, recent studies in Nile tilapia and Japanese sea bass also showed that water salinity significantly improved the contents of free amino acids and PUFA, such as EPA and DHA, in the muscle tissue [1,5,9,10]. Moreover, an experiment in marine euryhaline crab *Scylla paramamosain* showed that salinity had an impact on flesh hardness, chewiness, and gumminess, and the contents of flavor compounds in flesh [11]. With these results in mind, we speculated that rearing euryhaline fish species in proper salinity might be a practical approach to improving flesh quality, not only in the aspect of important nutrients such as EPA and DHA, but also in the aspects of texture and taste.

Texture profile analysis (TPA) and electronic-tongue (E-tongue) are effective methods assessing meat texture and taste substances, respectively, and have been widely used in livestock and poultry products analysis [12,13,14,15]. Recently, TPA and E-tongue have come into use for texture and taste analysis in aquatic animals [16,17]. Thus, it is feasible to assess the effect of salinity on flesh quality using TPA and E-tongue approaches.

Native to lakes and rivers in eastern North America, largemouth bass, *Micropterus salmoides*, has been widely introduced around the world. In China, for example, it is an important freshwater culture species. It is worth noting that largemouth bass is able to tolerate a wide range of salinity [18], and thus can be served as an ideal model to assess the effect of salinity on fish quality. Although higher energetic costs may lead to slower growth rate and smaller maximum size in largemouth bass [19,20], proper salinity may also improve fish flesh quality, contributing to the effect of salinity on the contents of critical nutrients and flesh texture and taste. The objective of this study is to evaluate the effect of rearing salinity on largemouth bass flesh quality, from the perspective of texture, taste and PUFA content.

## 2. Materials and Methods

### 2.1. Animal Ethics Approval

Our study on largemouth bass was approved by the Animal Care and Use Committee of the Yangzhou University (ethical protocol code: YZUDWSY 2017-09-06), and all efforts were made to minimize suffering. For example, experimental fish were anesthetized with MS-222 (150 mg/L, Sigma, St. Louis, MO, USA) before euthanizing and handling.

### 2.2. Fish and Experimental Conditions

Commercial largemouth bass fry, mean initial weight of 48.57 ± 0.11 g, was purchased from a farm in Zhejiang, China. The fish were fasted for 24 h, weighed, and randomly sorted into 9 tanks (300 L), with 25 fish in each tank. The fish were fed twice a day (2–3% of body weight) with commercial diet (crude protein 48, crude lipid 6.5, moisture 11, and ash 18) for 10 weeks. Water temperature was at 23~25 °C, and pH ranged from 7.7 to 8.3 during the feeding trial. Previous research reported that the growth rate of largemouth bass decreased with salinity up to 0.8% [20], so the maximum salinity in the present experiment was set at 0.9% (S_9_ group), which was supposed to impact not only growth performance but also flesh quality of largemouth bass. Moreover, a low salinity (0.3%, S_3_ group) was set according to our classification on saline-alkali land, where 0.3% is the upper threshold of mild saline–alkali land. Salinities of 0.3% and 0.9% were obtained by diluting artificial seawater prepared beforehand (Bosskas, Shanghai, China) and confirmed using a salimeter, while freshwater was treated as control (S_0_ group).

### 2.3. Sample Collection

Experimental fish were fasted for 24 h and anesthetized using MS222 before sampling. Ten fish from each tank were dissected for back muscle sampling.

### 2.4. Muscle Texture Analysis

#### 2.4.1. Drip Loss

After sampling, the muscle samples of each group were immediately trimmed into chunks of 10 ± 1.0 g (W_1_) and stored at 4 °C for 24 h. Then, Whatman quantitative filter paper (Φ 12.5 cm, WoHua filter paper Co., Ltd., Hangzhou, Zhejiang, China) was adopted to dry the samples before they were reweighed (W_2_). Drip loss was evaluated as (W_1_ − W_2_)/W_1_ × 100%.

#### 2.4.2. Cooking Loss

After drip loss measurement (W_1_), the samples were placed in new self-sealing polyethylene bags (thickness 0.12 mm, MingKe plastic industry Co., Ltd., Taizhou, Zhejiang, China) and cooked in a water bath of 80 °C for 30 min. Then, the samples were cooled in running water (15 °C) to room temperature, dried with filter paper, and reweighed (W_2_). Cooking loss was evaluated as (W_1_ − W_2_)/W_1_ × 100%.

#### 2.4.3. Centrifugal Loss

The muscle samples were weighted (W_1_, 10 ± 1.0 g), centrifuged at 4000 rpm and 4 °C for 30 min, and wrapped with filter paper. Then, the samples were reweighted (W_2_). Centrifugal loss was evaluated as (W_1_ − W_2_)/W_1_ × 100%.

#### 2.4.4. Shear Value

Shear force test was conducted according to Zhang et al., [21]. The muscle samples were stored at 4 °C for 24 h before being subjected to shear value determination using a Meat Tenderness Tester RH-N50 (Runhu Instrument Co., Ltd., Guangzhou, Guangdong, China). Parameters for the blade were as follows: thickness 3.0 ± 0.2 mm, height ≥ 35 mm, angle 60°. The sample was trimmed into two strips (1 × 4 cm), and each strip was cut four times. The mean of the eight cuts was taken as the final result of each sample.

#### 2.4.5. Texture Profile Analysis

Texture profile analysis (TPA) was performed according to Luo et al. [11] using a TMS-pro texture analyzer (FTC Food Technology Corporation, Sterling, VA, USA) equipped with a P/5 cylinder probe. Test parameters were set as follows: test speed 10 mm/min, test deformation 40% and trigger force 0.3 N. Flesh hardness, springiness, cohesiveness, gumminess, chewiness, and resilience were recorded.

#### 2.4.6. Morphology Analysis

The muscles were transversely cut into 1 × 1 × 1 cm^3^ blocks and fixed with fixative G1101 (Servicebio, Wuhan, Hubei, China) for 24 h, and then embedded in paraffin, sectioned and stained with hematoxylin-eosin (HE). After dehydration with alcohol and xylene and sealing with neutral gum, the samples were examined under ECLIPSE Ci-L microscope (NIKON Instrument, Shanghai, China). Then, electronic images were taken and analyzed using NIKON DS-U3 according to Luo et al. [11].

#### 2.4.7. Collagen Content Determination

Collagen content was estimated by hydroxyproline (Hyp) assay based on the observation that Hyp accounts for 13.4% of total collagen content [22]. The content of Hyp was determined using a commercial kit (Nanjing Jiancheng Bioengineering Institute, Nanjing, Jiangsu, China) according to the manufacturer’s instructions.

### 2.5. Identification and Quantification Taste Substances

#### 2.5.1. Fatty Acid Analysis

Total lipids were extracted from freeze-dried muscle samples (0.5~1 g) using Folch liquid (chloroform:methanol = 2:1 by vol.). Then, methanolic KOH was added, and the samples were blow dried with nitrogen before boron trifluoride and normal heptane were added sequentially. After the addition of saturated salt water, the supernatant was taken, and anhydrous sodium sulfate was added. Fatty acid methyl eaters were subjected to analysis using gas chromatography (GC-14B, Shimadzu, Suzhou, Jiangsu, China) with CP-Sil 88 column (0.25 mm × 50 m × 0.20 μm, Agilent Technologies, Beijing, China). A standard (47015-U, Supelco, Shanghai, China) was used to identify potential fatty acid components in each sample. Fatty acid composition (%) = 100 × (peak area of a certain fatty acid/peak area of total fatty acid).

#### 2.5.2. Nucleotide Analysis

Muscle samples (1 g~2 g) were homogenized in 6 mL of 6% perchloric acid by ultrasound. The extracts were centrifuged at 3500 rpm for 10 min at 4 °C before the supernatants were filtered through a 0.45 μm membrane. The pellet was re-extracted, and the supernatant was combined, diluted to 25 mL, adjusted to pH 6.5 ± 0.02, and analyzed by high performance liquid chromatography (HPLC, Agilent Technologies, Beijing, China) using a Diamonsil C18 (4.6 × 250 mm, 5μm) liquid chromatography column. Detection was performed in triplicate, and the results were expressed as mg/g fresh matter.

In addition, the taste impact of flavor nucleotides was evaluated by taste active value (TAV), i.e., the ratio of the concentration of a compound to its corresponding taste recognition threshold, and taste substances with TAV > 1 were considered as flavor contributors [23]. The greater the value, the greater the contribution. The TAV of each flavor nucleotide was calculated according to Song et al., [24].

#### 2.5.3. Free Amino Acids Analysis

Muscle samples were weighed (about 0.20 g) and digested with 10 mL HCl (0.02 mol/L) and homogenized for 45 s. The digested samples were resuspended in 1.5 mL HCl (0.02 mol/L) and filtered through a 0.22 μm membrane (CNW, Shanghai, China) to remove any residue and impurity. The filtrates were analyzed with automatic amino acid analyzer LA8080 (Hitachi, Shanghai, China). Sweet (Thr, Ser, Gly, Ala), umami (Asp, Glu), and bitter (His, Lys, Leu, Ile, Val) amino acids were defined according to Song et al., [24].

#### 2.5.4. Electronic Tongue

Five taste qualities were determined using electronic tongue (E-tongue), including umami, saltiness, sourness, bitterness, and astringency. In addition, the experiment included aftertaste bitterness (Aftertaste-B) and aftertaste astringency (Aftertaste-B). E-tongue analysis was conducted according to Buratti et al. [25] with some modifications. The fish mince samples (6~7 g) were put in valve bags for a water bath (40 °C) until the center of the mince reached 40 °C. Then, distilled water at a temperature of 40 °C was added in a ratio of 1:10, mixed, and centrifuged at 4000 rpm for 10 min, before the supernatant was measured using electronic tongue SA402B (Insent, Atsugi, Kanagawa, Japan). Fresh 30 mM KCl solution containing 0.3 mM tartaric acid was used as the reference (Vr) and to rinse the electrode. The electric potential of samples was defined as Vs, and the difference between Vs and Vr corresponds to the “taste immediately after putting in the mouth”. The electrodes were subsequently rinsed for 6 s and used to measure the potential of the reference again (Vr1). The difference between Vr1 and Vr corresponds to the “aftertaste”. Samples were measured in triplicate, and the mean of the three measurements was standardized and subjected to Principal Component Analysis.

### 2.6. Statistics, Calculations, and Statistical Analysis

Data normality and homoscedasticity were first checked before one-way ANOVA was adopted for data analysis, and differences shown in ANOVA were further analyzed using the Tukey’s method. Each result was presented as mean ± standard error. For principal component analysis (PCA), all data were homogenized and processed using GraphPad Prism 9 software (San Diego, CA, USA).

## 3. Results and Discussion

### 3.1. Muscle Texture Analysis

**Figure 1:**
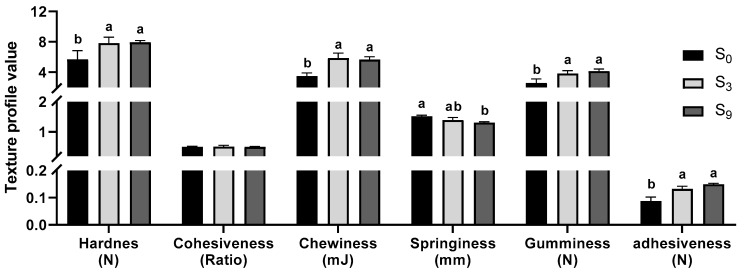
Effect of salinity on muscle texture profile in largemouth bass. Different lowercase letters indicate significant differences amone the treatment (*p* < 0.05). The same as below.

Shear value test revealed that salinity of 0.9% significantly decreased muscle tenderness compared with S_0_ group (*p* < 0.05, Table 1), which was consistent with the significantly decreased moisture content in 0.9% group in our previous study [26]. Similar results were also obtained in Japanese sea bass, in which marine cage culture led to a significant decrease in muscle moisture content and tenderness compared with freshwater pond culture [5]. Texture parameters, including hardness, chewiness, gumminess, and adhesiveness significantly increased with water salinity (*p* < 0.05, Figure 1) while springiness decreased (*p* < 0.05). These results support the abovementioned observation that muscle tenderness decreases with salinity.

On the other hand, the drip loss, cooking loss, and centrifugal loss were significantly decreased in S_3_ and S_9_ groups compared with S_0_ group (*p* < 0.05, Table 1), indicating that high water salinity resulted in high water-holding capacity in largemouth bass. It was concluded that a high rate of muscle water loss leads to protein loss along with water, and thus causes the muscle contraction and tenderness to decrease [27]. In other words, higher water-holding capacity resulting from salinity would help to maintain flesh tenderness in storage and cooking conditions.

**Figure 2:**
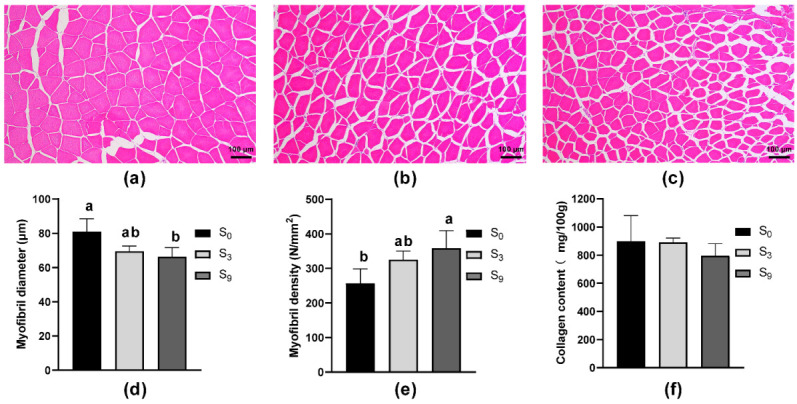
Effect of salinity on myofibril structure and collagen content of largemouth bass muscle. (**a**) Muscle histology sections of S_0_ group; (**b**) muscle histology sections of S_3_ group; (**c**) muscle histology sections of S_9_ group; (**d**) diameter of myofibers (μm) (*n* = 30, random 10 myofibers of every section, three sections of every group); (**e**) density of myofibers (N/mm^2^) (*n* = 30, random 10 myofiber of every section, three sections of every group); (**f**) collagen content in muscle (*n* = 3). All histology sections were photographed under 100× magnification. Bars with different letters (a, b) are significantly different (*p* < 0.05; Tukey’s test) between the two groups.

As a major component in intramuscular connective tissue, the content of collagens also affects muscle texture [11,28]. Here, we compared the content of proline plus hydroxyproline in the muscle of largemouth bass reared in different salinities, and found no significant difference (Figure 2), indicating that ambient salinity resulted in few effects on muscle collagen content. A similar result was also obtained in crab [11], in which the reduced flesh hardness, chewiness, and gumminess were attributed to altered myofibril structure. Inspired by this, we compared myofibril diameter and density in the samples, and revealed that high salinity significantly decreased myofibril diameter, but significantly increased myofibril density. Combined with the finding in our previous studies that salinity (or marine cage culture as compared with freshwater pond culture) significantly affects fish crude body composition [5,26], we conclude here that ambient salinity may change fish flesh texture through comprehensive effects on muscle composition and myofibril structure.

### 3.2. Muscle Taste Analysis

**Figure 3:**
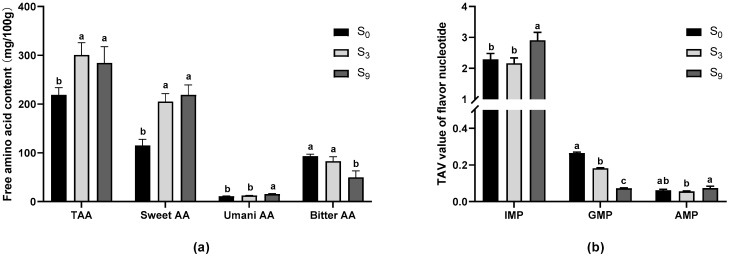
Effects of salinity on the the content of flavor substances in largemouth bass muscle. (**a**) The contents of free amino acid; (**b**) the content of flavor nucleotide. TAA is total free amino acid, Sweet AA is sweet amino acid, Umani AA is umani amino acid, and Bitter AA is bitter amino acid. Bars with different letters (a, b) are significantly different (*p* < 0.05; Tukey’s test) between the two groups.

Flesh taste is another attribute affecting fish quality. Fish muscle provides flavors including sweet, bitter, and umami due to abundant free amino acids and other taste substances [29]. Thus, factors that affect flavor amino acid content will impact on fish flesh taste, which in turn determines consumers’ choice. As a key environmental factor, salinity can greatly increase the content of total free amino acid in aquatic animals, which helps in keeping balance in osmoregulation [30,31]. Here, we report that salinity significantly increased the content of total free amino acid in largemouth bass (*p* < 0.05, Figure 3a), especially the contents of sweet amino acid and umami amino acid. In addition, the contents of umami amino acid (UAA) and bitter amino acid (BAA) in S_9_ group were significantly higher and lower than S_0_ group (*p* < 0.05), respectively. Similar results were also reported in Nile tilapia [1], kuruma shrimp [32], and mud crab [11]. It is worth noting that the effects of salinity on free amino acid content are not entirely uniform across aquatic animals. For example, in Nile Tilapia, ambient salinity significantly increased the contents of eight out of the 17 detected amino acids, including glycine, alanine, glutamate, proline, aspartate, tyrosine, arginine, and isoleucine [1]. In kuruma shrimp, however, ambient salinity brought no significant difference in the contents of glycine and arginine [32]. Similarly, salinity of 0.9% significantly decreased bitter amino acid content in largemouth bass in the present study, while higher salinity significantly increased the content of bitter amino acid in mud crab [11]. This inconsistent change in the content of free amino acid in response to ambient salinity might reflect different responses and metabolic regulation mechanisms under salinity stress in different aquatic animals. Given that these experiments were conducted by different researchers under different conditions, it may also be due to other factors, such as the nutritional status of the experimental animals, etc.

Moreover, 5′-nucleotides also contribute to the umami taste of fish flesh. We determined the contents of AMP, GMP, and IMP in the muscle of largemouth bass, and found that only the TAV value of IMP was greater than one, irrespective of water salinity (Figure 3b), indicating that IMP is a significant taste contributor in largemouth bass. The dominant flavor nucleotides are different in the muscles of different aquatic animals. For instance, the main flavor nucleotides in Pacific white shrimp are AMP and IMP [33], and in minced sturgeon the main flavor nucleotide is IMP [34]. In Antarctic krill, however, the contents of AMP, IMP, and GMP are all low [33]. Moreover, salinity of 0.9% significantly increased the content of IMP compared with the control group (*p* < 0.05, Figure 3b), which would enhance the umami taste further. Although salinity of 0.9% significantly decreased the content of GMP in the muscle of largemouth bass, it had little effect on the flesh taste because of its low TAV. This result is comparable with the finding in mud crab, in which lower salinity significantly reduced the content of AMP, the dominant flavor nucleotide [11]. Our previous study in Japanese sea bass also showed that seawater cultured fish had significantly higher IMP content than fish reared in freshwater ponds (unpublished). As a more potent flavor enhancer, the contribution of IMP to meat flavor has been well determined in livestock and poultry [35,36,37]. Hence, it seems obvious that salinity tends to improve flesh taste through promoting the content of IMP in largemouth bass.

**Figure 4:**
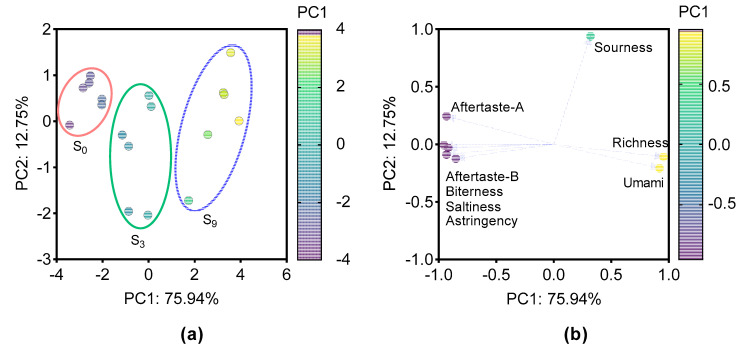
PCA from E-tongue data of largemouth seabass muscle with different salinity. (**a**) Score plot; the three groups are circled by different ovals; (**b**) loading plot. The color of each point represents the score along the PC1 direction.

Complex interactions among various compounds affect the perception of meat flavor. Thus, we further analyzed the comprehensive effect of rearing salinity on flesh taste using E-tongue method followed by PCA analysis (Figure 4). The variance contribution rates of the PC1 and PC2 were 75.9% and 12.7%, respectively, accounting for over 88% variance contribution rates in total. Figure 4a clearly shows that the three groups can be easily discriminated along the PC1 direction, while a large difference exists in the groups S_3_ and S_9_ along the PC2 direction. On the other hand, most of the tastes detected together with taste richness mainly contributed to inter-group difference along the direction of PC1, and sourness mainly contributed to difference along the direction of PC2. In addition, the umami tastes and taste richness potentially contributed further to the sample S_9_, while the tastes of saltiness, bitterness, astringency, and aftertaste-B potentially contributed further to the sample S_0_. This was consistent with the results for free amino acid and nucleotide, two critical flavor substances determining meat taste. Therefore, it was obvious that water salinity raised the contents of sweet and umami free amino acid and IMP in the muscle, resulting in the taste richness and umami taste being enhanced in S_9_ group.

### 3.3. Fatty Acids Composition

As an important nutrient, n-3 LC-PUFA plays important roles in many biological processes in vertebrates [38]. In the present research, the contents of most fatty acids detected in muscle tissue were affected significantly by water salinity (Table 2), among which the contents of MUFA and n-6 PUFA in the S_3_ and S_9_ groups were significantly lower than S_0_ group (*p* < 0.05), while the contents of n-3 LC-PUFA, including C20: 5n-3 (EPA) and C22: 6n-3 (DHA), and the ratio of n-3 PUFA to n-6 PUFA were significantly higher in S_3_ and S_9_ groups than S_0_ group (*p* < 0.05). In fact, salinity promotes deposition of EPA, and DHA has been well-verified in several fish species [5,39,40]. Therefore, it seems a practical approach to rearing fish in water with proper salinity to enhance EPA and DHA deposition from the perspective of human nutrition.

## 4. Conclusions

Our study clearly showed that ambient salinity brought out significant effects on largemouth bass flesh quality. Although muscle tenderness decreased with salinity level, other indexes, such as water-holding capacity, umami taste, and the content of n-3 LC-PUFA, were significantly improved. This is of great significance for improving the flesh quality of euryhaline fish species through modifying rearing conditions. As salinity may also lead to decreased fish growth performance, however, it is critical to further determine the appropriate salinity level and the shortest culture period that is necessary for improving flesh quality.

## Figures and Tables

**Table 1:** Effects of salinity on water loss and shear value of muscle in *M. salmoides* (means ± S.D., *n* = 3).

Index	S_0_	S_3_	S_9_
Drip loss, %	5.12 ± 0.33 ^a^	3.96 ± 0.32 ^b^	3.40 ± 0.24 ^b^
Cooking loss, %	22.24 ± 0.27 ^a^	18.66 ± 1.34 ^b^	19.07 ± 0.69 ^b^
Centrifugal loss, %	17.73 ± 0.27 ^a^	15.28 ± 0.12 ^b^	14.17 ± 0.38 ^b^
Shear value, N	4.74 ± 0.12 ^b^	5.28 ± 0.22 ^ab^	6.19 ± 0.34 ^a^

Different lower-case letters (a, b) in a row indicate significant differences (*p* < 0.05) between the two groups.

**Table 2:** Effects of salinity on fatty acid contents of muscle in *M. salmoides* (%, means ± S.D., *n* = 3).

Fatty Acid ^1^	S_0_	S_3_	S_9_
C14:0	1.98 ± 0.10	1.59 ± 0.40	1.38 ± 0.15
C16:0	16.86 ± 0.97	20.12 ± 1.80	20.03 ± 0.99
C18:0	3.11 ± 0.21 ^b^	5.05 ± 1.39 ^ab^	7.32 ± 1.24 ^a^
∑SFA ^2^	21.95 ± 1.03 ^b^	26.75 ± 2.95 ^ab^	28.74 ± 1.50 ^a^
C16:1	4.71 ± 0.23 ^a^	2.63 ± 0.23 ^b^	1.90 ± 0.15 ^c^
C18:1	25.48 ± 0.89 ^a^	21.96 ± 2.31 ^a^	17.51 ± 1.42 ^b^
∑MUFA ^3^	30.19 ± 0.98 ^a^	24.59 ± 2.40 ^b^	19.40 ± 1.55 ^c^
C18:2n−6	32.13 ± 2.55 ^a^	25.77 ± 1.56 ^b^	25.70 ± 1.67 ^b^
C20:4n−6	0.89 ± 0.09 ^b^	1.26 ± 0.27 ^b^	2.01 ± 0.19 ^a^
∑n−6 PUFA ^4^	33.02 ± 2.57 ^a^	27.03 ± 1.40 ^b^	27.72 ± 1.82 ^b^
C18:3n−3	4.59 ± 0.39 ^a^	3.49 ± 0.46 ^b^	2.74 ± 0.09 ^b^
C20:5n−3	1.06 ± 0.13 ^b^	1.73 ± 0.13 ^a^	1.86 ± 0.19 ^a^
C22:6n−3	7.14 ± 1.52 ^b^	14.98 ± 2.0 ^a^	19.18 ± 1.71 ^a^
∑n−3 PUFA ^5^	12.80 ± 1.21 ^b^	20.20 ± 1.67 ^a^	23.78 ± 1.80 ^a^
∑n−3/∑n−6 PUFA	0.39 ± 0.07 ^b^	0.75 ± 0.09 ^a^	0.86 ± 0.12 ^a^
∑n−3 LC-PUFA	8.21 ± 0.10 ^b^	16.71 ± 2.12 ^a^	21.05 ± 1.89 ^a^

^1^ Some fatty acids that were present in only minor or trace amounts or that were not detected, such as C22:0, C24:0, C14:1, C20:2n−6, and C20:3n−6, are not listed in the table. For fatty acid detect limit in the present study, please find Table S1 in Supplementaty Material. ^2^ SFA: Saturated fatty acid. ^3^ MUFA: Monounsaturated fatty acid. ^4^ n−6 PUFA: n−6 poly-unsaturated fatty acid. ^5^ n−3 PUFA: n−3 poly-unsaturated fatty acid. Different lower-case letters (a, b) in a row indicate significant differences (*p* < 0.05) between the two groups.

## Data Availability

The data presented in this study are available on request from the corresponding author.

## Supplementary Materials

The following supporting information can be downloaded at: https://www.mdpi.com/article/10.3390/foods11203261/s1, Table S1: Detect limit of fatty acids in the present study.
