# Supplementary material for: The Impact of Rearing Salinity on Flesh Texture, Taste, and Fatty Acid Composition in Largemouth Bass Micropterus salmoides
Source: Foods. 2022 Oct 19;11(20):3261. doi: 10.3390/foods11203261 (PMC9601509; doi:10.3390/foods11203261)
Supplement: Supplementary file 1 [file foods-11-03261-s001.zip › foods-1948611-supplementary.pdf]

foods

MDPI

Table S1. Detect limit of fatty acids in the present study.

Fatty acids	Detect limit（mg/Kg）	Fi	Fj
C8:0	0.5	0.9114	0.9192
C10:0	0.5	0.9247	0.9314
C11:0	0.5	0.9300	0.9363
C12:0	0.5	0.9346	0.9405
C13:0	0.5	0.9386	0.9442
C14:0	0.5	0.9421	0.9473
C14:1n5	0.5	0.9417	0.9470
C15:0	0.5	0.9453	0.9502
C15:1n5	0.5	0.9449	0.9499
C16:0	0.5	0.9481	0.9529
C16：1n7	0.5	0.9477	0.9525
C17:0	0.5	0.9507	0.9552
C17:1n7	0.5	0.9503	0.9549
C18:0	0.5	0.9530	0.9573
C18：1n9t	0.5	0.9527	0.9570
C18：1n9c	0.5	0.9527	0.9571
C18：2n6t	0.5	0.9524	0.9568
C18：2n6c	0.5	0.9524	0.9568
C20:0	0.5	0.9570	0.9609
C18:3n6	0.5	0.9520	0.9559
C20:1	0.5	0.9568	0.9608
C183n3	0.5	0.9520	0.9560
C21:0	0.5	0.9588	0.9628
C20:2	0.5	0.9565	0.9605
C22:0	0.5	0.9604	0.9642
C20:3n6	0.5	0.9562	0.9598
C22:1n9	0.5	0.9602	0.9639
C20:3n3	0.5	0.9562	0.9598
C20:4n6	0.5	0.9560	0.9597
C23:0	0.5	0.9620	0.9658
C22:2n6	0.5	0.9600	0.9638
C24:0	0.5	0.9963	1.0002
C20:5n3	1.0	0.9557	0.9592
C24:1n9	1.0	0.9632	0.9666
C22:6n3	1.0	0.9590	0.9624

Note: Fi represent conversion factor of fatty acid methyl ester to fatty acid, Fj represent conversion factor of fatty acid triglycerides to fatty acids.
